# Structure of vaccinia virus thymidine kinase in complex with dTTP: insights for drug design

**DOI:** 10.1186/1472-6807-6-22

**Published:** 2006-10-24

**Authors:** Kamel El Omari, Nicola Solaroli, Anna Karlsson, Jan Balzarini, David K Stammers

**Affiliations:** 1 Division of Structural Biology, The Wellcome Trust Centre for Human Genetics, University of Oxford, Oxford OX3 7BN, UK; 2 Karolinska Institute, Huddinge University Hospital, S-141 86, Stockholm, Sweden; 3 Rega Institute for Medical Research, K.U. Leuven, B-3000 Leuven, Belgium

## Abstract

**Background:**

Development of countermeasures to bioterrorist threats such as those posed by the smallpox virus (variola), include vaccination and drug development. Selective activation of nucleoside analogues by virus-encoded thymidine (dThd) kinases (TK) represents one of the most successful strategies for antiviral chemotherapy as demonstrated for anti-herpes drugs. Vaccinia virus TK is a close orthologue of variola TK but also shares a relatively high sequence identity to human type 2 TK (hTK), thus achieving drug selectivity relative to the host enzyme is challenging.

**Results:**

In order to identify any differences compared to hTK that may be exploitable in drug design, we have determined the crystal structure of VVTK, in complex with thymidine 5'-triphosphate (dTTP). Although most of the active site residues are conserved between hTK and VVTK, we observe a difference in conformation of residues Asp-43 and Arg-45. The equivalent residues in hTK hydrogen bond to dTTP, whereas in subunit D of VVTK, Asp-43 and Arg-45 adopt a different conformation preventing interaction with this nucleotide. Asp-43 and Arg-45 are present in a flexible loop, which is disordered in subunits A, B and C. The observed difference in conformation and flexibility may also explain the ability of VVTK to phosphorylate (South)-methanocarbathymine whereas, in contrast, no substrate activity with hTK is reported for this compound.

**Conclusion:**

The difference in conformation for Asp-43 and Arg-45 could thus be used in drug design to generate VVTK/Variola TK-selective nucleoside analogue substrates and/or inhibitors that have lower affinity for hTK.

## Background

Thymidine kinases form part of the salvage pathway for pyrimidine deoxyribonucleotide synthesis. TKs are expressed in a variety of organisms from human to bacteria as well as in a number of viruses. The reaction catalysed by TK involves the transfer of a γ-phosphoryl moiety from ATP to 2'deoxy-thymidine (dThd) to produce thymidine 5'-monophosphate (dTMP). Certain TKs, such as those from herpes simplex virus type 1 (HSV-1) and varicella zoster virus (VZV) have, in addition, thymidylate kinase activity allowing the conversion of dTMP to thymidine 5'-diphosphate (dTDP). TKs can be classified into two types which differ in several respects [1]. Type 1 TKs are of higher molecular weight, typically around 40 kDa, and are active as homodimers. This subfamily contains the HSV1, HSV2 and VZV TKs, and also mitochondrial TK.

TKs of type 2 include those from poxviridae such as vaccinia virus (VV) and variola virus, [2], as well as from human [3] hTK, (human type II thymidine kinase 1) and mouse [4]. Type 2 TKs have a smaller polypeptide chain compared to type 1, being of ~25 KDa but form homotetramers. They are sensitive to the feedback inhibitors dTDP or dTTP, which are generated at the end of the metabolic pathway [5]. Type 2 TKs have a much narrower substrate specificity compared to type 1 TKs and only phosphorylate 2'deoxyuridine (dU) and/or dThd [6]. For example, the antiherpetic drug (*E*)-5-(2-bromovinyl)-dUrd (BVDU) [7] is not metabolised by the type 2 TKs (i.e. cytosolic TK) in contrast to the type 1 TKs (i.e. mitochondrial TK, HSV-1 TK) which can phosphorylate a variety of (5-substituted) nucleoside analogues including BVDU. Moreover, HSV-1 and HSV-2 TK can even recognize (acyclic) purine nucleoside analogues such as acyclovir and ganciclovir [8]. This difference in substrate specificity is the basis of some selective antiviral drug action as validated by the activation of nucleoside analogues by certain herpes virus TKs. Moreover, herpes TKs are also being studied as suicide genes in a combined gene/chemotherapy strategy to treat cancer [9].

The World Health Organisation declared in 1980 that smallpox had been eradicated. Since then, routine inoculation with the vaccinia virus vaccine was discontinued, resulting in minimal or even non-existent smallpox immunity in the human population [10]. Today, the potential use of smallpox virus as a biological weapon is a major cause for concern, particularly in the context of current low levels of herd immunity to the virus. Additionally, the re-emergence of monkeypox virus infection in humans (mainly in Africa but some cases have also been reported in the United States [11]), has lead to the stockpiling of smallpox vaccine (VV), mainly in developed countries [12]. Nevertheless, some adverse reactions which are sometimes lethal, following vaccination have been reported [13-15]. VV should neither be given to pregnant women for example, nor to people who have a weakened immune system, skin problems like eczema, heart problems or to children under one year old [12]. Thus, specific anti-variola drugs need to be developed as a matter of priority, particularly for widespread use in a bioterrorism emergency, as well as for specific cases of unwanted contamination by VV or complications like eczema vaccinatum or progressive vaccinia. Such drugs would be of particular importance if more virulent strains of variola virus were engineered using genetic modifications. Worryingly, one of the only anti-variola drugs available, cidofovir [16], has been reported to be inefficient against several pox viral strains [17].

Herpes virus TKs have been exploited in the selective activation of nucleoside analogues such as acyclovir, which have spawned a series of highly successful anti-herpetic drugs [18]. The same approach may therefore be applicable in the case of orthopoxviruses. Further understanding of the structural differences between type 1 and 2 TKs would also greatly help to improve and/or create specific drugs against the type 1 TK. Indeed, a drug would be selective if it is preferentially metabolised by the type 1 and not the type 2 TKs or *vice versa*. Thus gathering structural information about both TK types is likely to be of great importance in assisting drug design. So far, HSV1 TK [19,20] and VZV TK [21] structures have been solved, whilst for type 2 TK, the structure of human cytosolic thymidine kinase [22,23] and the TK of *Ureaplasma ureolyticum *recently became available [22,24]. Moreover, determining the structure of different type 2 TKs could help as in the case of orthopox viruses, to design virus TK-specific drugs. Recently, 5-(2-amino-3-cyano-5-oxo-5,6,7,8-tetrahydro-4*H*-chromen-4-yl)-1-(2-deoxy-pentofuranosyl)pyrimidine-2,4-(1*H*,3*H*)-dione has been shown to be phophorylated specifically by vaccinia and cowpox virus TKs [25].

We report here for the first time, the structure of VVTK, one of the smallest type 2 TKs known, in complex with dTTP at 3.1 Å resolution. This work will be of use, in combination with the previous type 2 TK structures, to design or improve type 1 TK-selective drugs as well as drugs that show selectivity against orthopox virus TKs such as VVTK or Variola TK.

## Results and discussion

### VVTK enzyme kinetics

**Figure 1:**
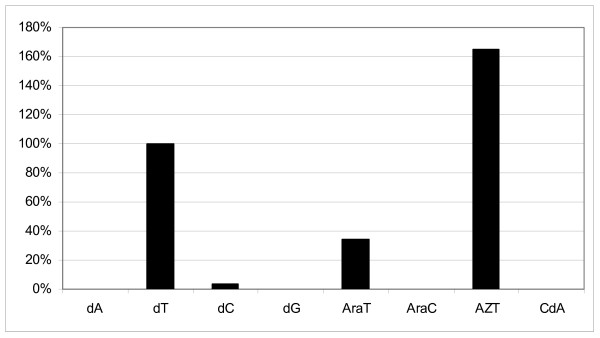
Histogram showing substrate activity of certain nucleosides with VVTK.

Assessment of the activity of VVTK to phosphorylate various nucleosides showed its ability to recognize both dCyd and dThd amongst the natural substrates. The efficiency of dCyd phosphorylation was, however, much less (<5%) compared to that with dThd (Fig. 1). In contrast, purified cytosolic TK showed a poor, if any, recognition of dCyd (data not shown). VVTK also efficiently converted the thymidine analogues araT and AZT to their corresponding mono-phosphates whereas no substrate activity was observed for the purine nucleosides araA, dA, dG and CdA. In this respect, VVTK behaves more similarly to cytosolic TK (Fig. 1). Also, dTTP inhibits the phosphorylation of dThd (1 μM) by VVTK with an IC_50 _(50% inhibitory concentration) of 14 ± 4.0 μM, whereas the corresponding IC_50 _for cytosolic TK was markedly lower (2.8 ± 0.5 μM).

### Overall structure of VVTK

**Figure 2:**
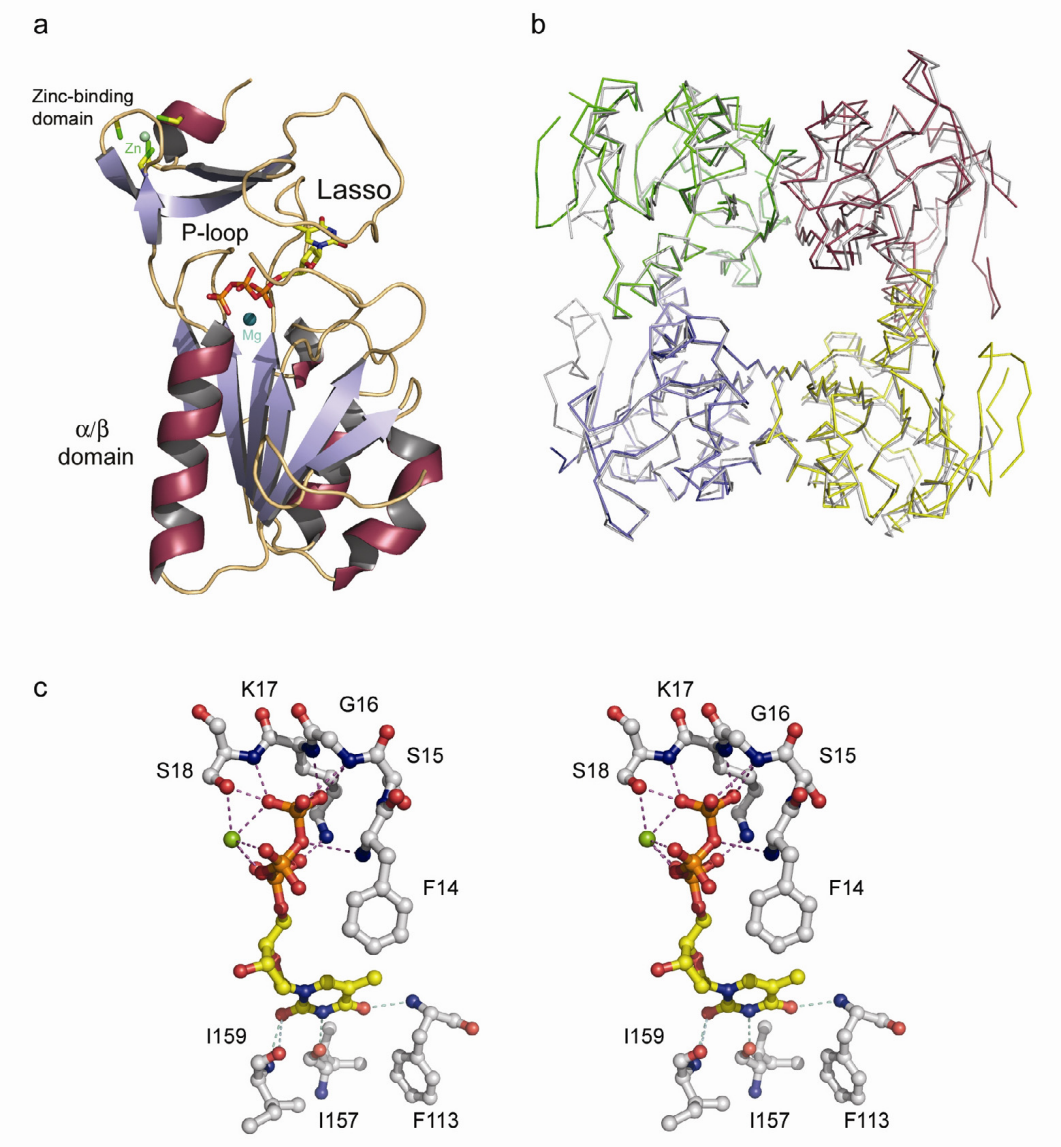
**a**. Diagram showing one VVTK monomer complexed with dTTP and magnesium. α-helices are shown in red/grey, β-strands are in blue/grey with loops in gold. dTTP is in standard atom colors and the magnesium ion is in blue. **b**. Diagram showing the superimposition of α-carbons of hTK (grey) and VVTK. Each VVTK subunit is shown in different colors (green/red/yellow/blue). **c**. Stereo-diagram of the VVTK active site showing residues that interact with dTTP and magnesium. Protein side-chains and main-chain carbon atoms are in grey, main-chain N and O atoms are in blue and red respectively). dTTP is drawn in standard atom colors with the magnesium ion in green

The quaternary structure of VVTK (Fig. 2a and 2b) is tetrameric. which is similar to that of hTK [22,23]. hTK shares 66% amino acid sequence identity with VVTK, showing a conserved fold amongst species (Fig. 2b). The four VVTK subunits within the tetramer are identical apart from a flexible loop (residues 40 to 60) situated on the surface. In the other type 2 TK structures [22-24], the equivalent loop is either not visible or appears to be in a different conformation from one monomer to the other, indicating that this flexibility is common to Type 2 TKs. It has been inferred that this loop might be involved in phosphoryl donor interaction [24].

As found in other type 2 TKs, VVTK has a central α/β domain structure consisting of six parallel β-strands surrounded by helices (Fig. 2a). Above the α/β domain, a zinc-binding domain is present, the latter belongs to the structural zinc-binding class [26]. Although no zinc was added during purification or crystallization, the metal is present in the structure and coordinated to four cysteines. This metal is believed to play a structural role, stabilising the adjacent loop, or lasso domain, involved in nucleoside binding via residues Tyr-166 and Arg-150. The cysteines involved in zinc binding are also found in the hTK, whilst in the case of the *Ureaplasma *enzyme, the last cysteine is substituted by a histidine [22].

### Active site structure of VVTK

**Figure 3:**
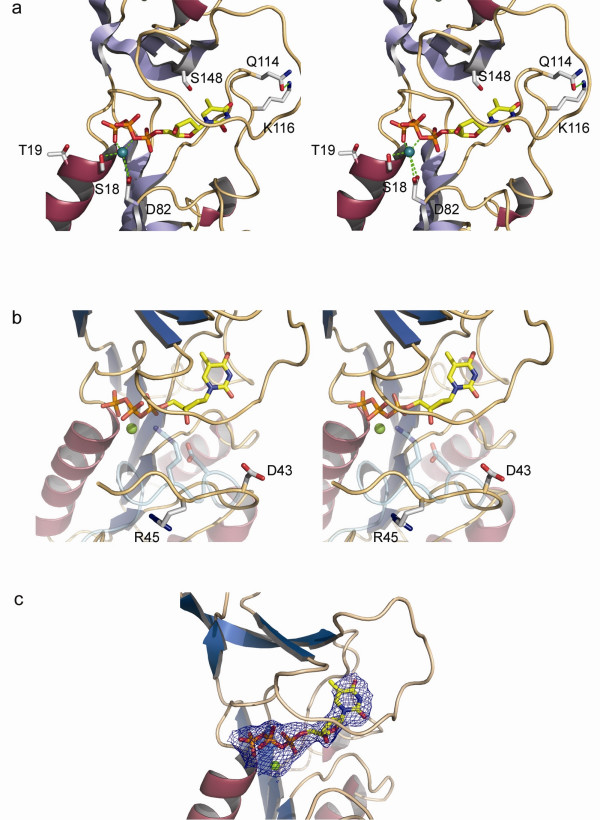
**a**. Stereo-diagram highlighting the positions ofresidues studied in site-directed mutagenesis experiments of VVTK. Side chains subjected to mutation are shown in grey. **b**. Stereo-diagram showing the different conformations of residues Asp-43 and Arg-45 in subunit D of VVTK and the equivalent hTK residues colored in cyan. **c**. Diagram showing the final 2fo-fc map contored at 1σ for dTTP and magnesium. Color coding for the protein secondary structure is as for Fig 2a.

VVTK crystals grew in the presence of dTTP, the feedback inhibitor of the pathway. The binding of dTTP in VVTK is similar to that observed in the hTK structure. The phosphates of dTTP bind to the α/β domain, whereas the thymine base and the deoxyribose bind between the lasso domain and the α/β domain (Figs. 2a, 3c). The thymine moiety is bound to the backbone of the protein via residues Phe-113, Ile-157 and 159. Thus this region of the enzyme appears unsuitable for use in designing species-selective ligands as no side-chain contacts are present (Fig. 2c).

Whilst most of the VVTK active site residues are conserved and superimpose well with the human TK, nevertheless, some significant differences are apparent. In the VVTK active site Ser-148 replaces the equivalent residue in hTK, Thr-163, a serine being also present in UuTK. Welin *et al*, proposed that this position, relatively close to the 5-methyl group of dTTP, could be used in the design of selective nucleoside analogues for UuTK versus the human enzyme. Indeed the pocket around the 5-methyl group of dTTP is slightly larger compared to the pocket of hTK, so substitutions at the 5 position of dThd could be explored [24]. Based on the same argument, these kinds of analogues may also be effective with VVTK and UuTK but not hTK.

A further important difference in the VVTK active site is the conformation of Asp-43 and Arg-45 (Fig. 3b). The equivalent residues in hTK are in close contact with the 3'-oxygen of the deoxyribose and to the β-phosphoryl oxygen of dTTP respectively, whereas they are not in the case of VVTK. Asp-43 and Arg-45 are only observed in the VVTK subunit D, they are disordered in the other three subunits, indicating the flexible nature of this loop. Nevertheless, the dTTP still adopts the same conformation in each subunit as observed in the human enzyme. We thus can infer that Asp-43 and Arg-45 may not be crucial for the binding of dTTP, otherwise this ligand would either not stay bound to the active site or would shift position. It has been proposed that Arg-60 in hTK (Arg-45 in VVTK) may act to stabilize the transition state of the reaction [22]. From the VVTK structure, it appears that the conformation of this residue has to change to allow contact with the substrate and hence the reaction to occur. The flexibility of this loop region also implies that more space is available in the VVTK active site compared to hTK. Clearly, using bulkier groups at the 3- and 5-positions in the pyrimidine ring of the dThd molecule should be investigated for the rational design of novel, specific drugs against VVTK. Modifying the sugar moiety would also be a worthwhile approach to pursue in the design of further analogues.

### Ligand specificity of VVTK

**Figure 4:**
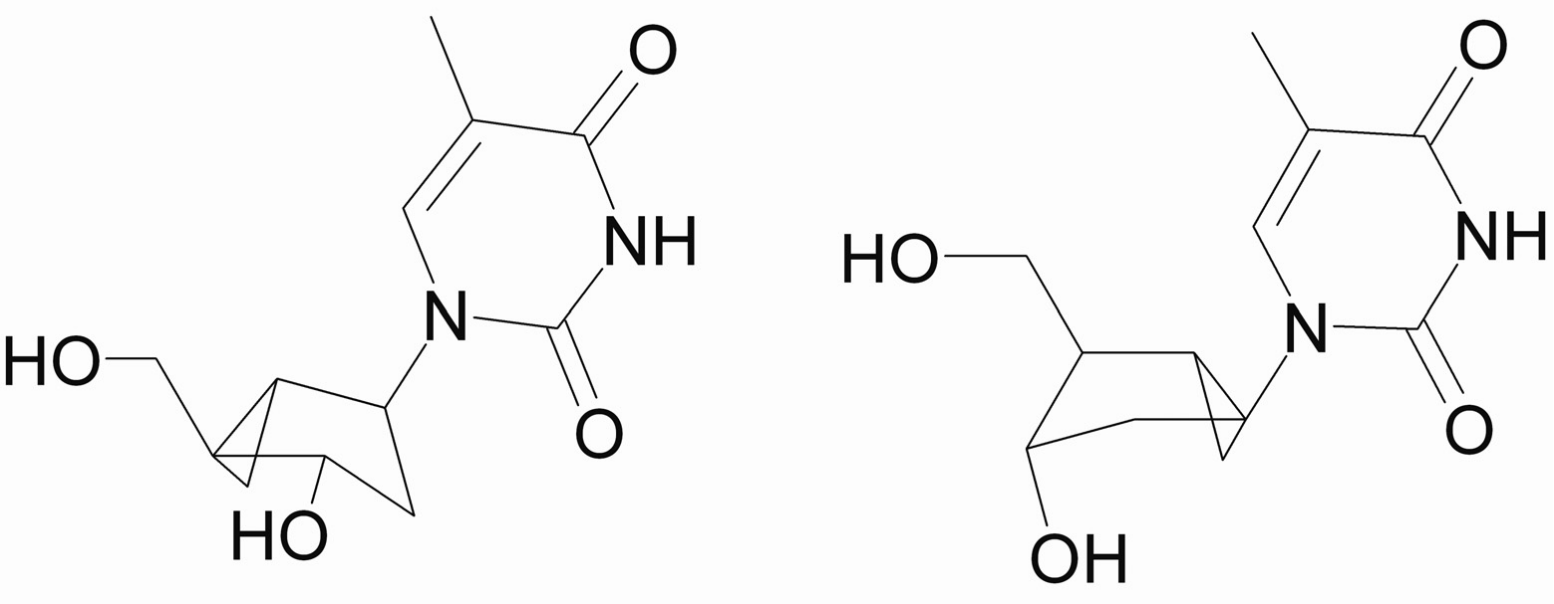
Structure of (north)-methanocarbathymine (left) and (south)-methanocarbathymine (right).

Thymidine analogues such as (South)-methanocarbathymine ((S)-MCT) and (North)-methanocarbathymine ((N)-MCT) (Fig. 4) have been studied as antiviral agents against orthopox and herpes viruses. For orthopox viruses, such as variola and vaccinia viruses, ((N)-MCT has potent antiviral activity whilst ((S)-MCT) does not [27,28]. Due to the pseudo-ring conformation, ((S)-MCT) has been reported to be the preferred substrate of herpes TKs, whereas ((N)-MCT) is preferred by the cellular DNA polymerases [29]. Prichard *et al *suggested that ((N)-MCT) could also be phosphorylated by type 2 TKs from cowpox and vaccinia viruses [28], whereas Smee *et al *inferred that orthopoxvirus TKs are not responsible for the formation of phosphorylated ((N)-MCT) [27]. It has also been shown that hTK has a weak affinity for ((N)-MCT) and essentially no affinity for ((S)-MCT) [29]. ((S)-MCT) inhibits growth of HSV1 TK transduced osteosarcoma cells [30], thus hTK alone could not be involved in drug activation. The MCT activity seems to be highly dependent on the particular cell line, which is thus a consideration in validating a particular result. Thus the test system ultimately of most relevance to human therapy would involve primates [27].

Nevertheless, HPLC activity assays have shown that ((S)-MCT) can be phosphorylated by VVTK [23], thus MCT modeled in the VVTK active site structure could be used as a basis for drug design. The superimposition of (S)-MCT and dTTP shows that the thymine ring and the sugar/pseudo-sugar match very well. As the (S)-MCT can be phosphorylated, this suggests that the conformation of the dThd moiety of dTTP has a very similar conformation to the dThd substrate in VVTK. This has been confirmed by the recent structure of UuTK complexed with dThd (PDB code: 2B8T) [24]. The fact that VVTK is able to phosphorylate (S)-MCT suggests that a larger sugar moiety can fit into the active site. Such a difference in substrate specificity between hTK and VVTK might be due to the flexibility of the loop containing Arg-45 (Arg-60 in hTK).

Further suggestion that the plasticity of the VVTK active site relative to hTK is indicated by the selective phosphorylatioin by VVTK compared to hTK of the bulky molecule 5-(2-amino-3-cyano-5-oxo-5,6,7,8-tetrahydro-4*H*-chromen-4-yl)-1-(2-deoxy-pentofuranosyl)pyrimidine-2,4-(1*H*,3*H*)-dione. The latter drug showed a minimal toxicity to uninfected cells and inhibited the replication of vaccinia and cowpox virus [25]. Fan *et al*, suggested that orthopoxvirus kinases are more promiscuous than previously believed. Indeed their substrate specificity seems broader than cellular kinases, thus these enzymes could be exploited in a similar manner to the herpes TKs.

### Relating VVTK mutagenesis and structural studies

Prior to the determination of any crystal structure of a type 2 TK, some mutational studies had been reported on VVTK. It has been shown that residue Asp-82 participates in the binding of magnesium [31] (Fig. 3a) and that the loss of a negatively charged residue at this position alters the ability of VVTK to transfer the γ-phosphate moiety of ATP to dThd. It was suggested that Asp-82 is not involved in ATP binding but rather aids its correct orientation for nucleophilic attack. In the VVTK structure, Asp-82 is indeed orientated towards the metal ion and its distance from the magnesium ion varies from one subunit to another (from 3.2 to 3.7 Å).

VVTK mutants Ser18Thr and Thr19Ser (Fig. 3a) [32] have also been studied, and showed an increased activity of 3.7-fold and 1.4-fold, respectively. The equivalent to Thr-19 is conserved in most type 2 TKs although not in the case of *Ureaplasma*, where it is substituted by an alanine and thus is unlikely to be essential for catalysis. Indeed, VVTK mutated in this position to the bulkier residues Asn or Arg retains significant activity (~27% of wild-type). Even a Pro-19 mutant retains 10% of wild-type VVTK activity. Thr-19 is not directly involved in dTTP binding but may have a role in ATP binding. As yet no type 2 TK has been solved with bound ATP, hence it is difficult to assess the importance of this residue. The Ser18Thr mutant shows a greater enhancement of enzyme activity (~3.8-fold) than does Thr19Ser and indeed in our structure, Ser-18 forms hydrogen bonds via its main-chain nitrogen to a γ-phosphate oxygen and via its side-chain oxygen to the magnesium and a γ-phosphate oxygen. Thus Ser-18 would appear more crucial for enzyme catalysis than Thr-19. Although Ser-18 is conserved in the hTK structure (Ser-33) it is conservatively substituted for threonine in UuTK (Thr26). The methyl group of the Thr-26 side-chain is in van der Waals contact with Ile-30 (3.8 Å). It is thus possible that replacing the serine by threonine could stabilize the side-chain oxygen in a preferential conformation for substrate binding, which may explain the increased activity of the Ser18Thr mutant VVTK.

Finally, mutational studies have been reported on Gln-114 [33] (Fig. 3a). The authors inferred that Gln-114 participates in the feedback inhibition by dTTP since this mutant TK could bind dTTP yet feedback inhibition was abolished. Interestingly, Gln-114 is not in contact with dTTP, and more surprisingly, it is located on the surface of the tetramer. The effect of mutating Gln-114 to either His or Asp would only be to break the hydrogen bond with the adjacent Lys. In this case, the abolition of the feedback inhibition could only be explained by a destabilization of the loop which is in close contact with the dTTP molecule. However, the mutation does not apparently affect the VVTK activity under standard conditions, thus either the binding of substrates dThd and ATP are not disrupted by this mutation, or if they are, the decrease in apparent affinity is compensated for as a result of an increase in catalytic rate. To clarify this, further experiments would be needed to determine the k_cat _and K_m _of each mutant.

## Conclusion

In conclusion, the work reported here provides the first structure of VVTK. Even though showing similarity to the other type 2 TK structures, the detailed comparison of the active site points to principles for designing specific inhibitors of VVTK relative to hTK, mainly by using the fact that the deoxyribose appears able to accept bulkier/modified substituents. This result is consistent with Birringer *et al*'s report of (S)-MCT being phosphorylated by VVTK whilst not being a substrate for hTK [23]. Knowledge of the residues involved in ligand binding of type 2 TKs by structure comparison, might also assist in the design of specific drugs against type 1 TKs.

## Methods

### Protein purification

The pET-16b-VVTK (derived from Novagen pET-16b) expression plasmid coding for an N-terminal His_6_-tagged-VVTK was transformed into Rosetta(DE3)pLysS for protein expression. The cells were grown in a 40 ml Luria Broth starter culture supplemented with 50 μg/ml carbenicillin and 34 μg/ml chloramphenicol, overnight, with shaking at 37°C. The culture was then diluted 1/100 into 4 l of LB and grown at 37°C until the *A*_600 _reached 0.7. isopropyl-beta-D-thiogalactopyranoside was added to 1 mM and the induction was carried out for 3 hours at the same temperature. The cells were harvested by centrifugation and the pellets were resuspended in buffer A (50 mM sodium inorganic phosphate pH 7.8, 300 mM NaCl). The cells were disrupted by sonication and the supernatant, following clarification by centrifugation, was applied to a 5 ml nickel chelating column (Hi-trap chelating Column, GE Healthcare) pre-equilibrated with buffer A. VVTK was eluted with buffer A + 500 mM imidazole. Fractions containing VVTK were applied to a Superdex S200 16/60 gel filtration column pre-equilibrated with 50 mM Tris-HCl pH 7.8 and 200 mM NaCl. The fractions containing VVTK were pooled and buffer exchanged against 20 mM Tris-HCl pH 8 and finally, concentrated to 22 mg/ml. The purification protocol produced on average 25 mg of VVTK per litre of culture.

### Crystallisation and data collection

Thymidine triphosphate, dithiothreitol and MgCl_2 _were added to VVTK for initial screening in several Hampton, Wizard and Emerald kits in a total of 768 conditions. A Cartesian Technologies pipetting robot was used to set up 100 + 100 nl sitting drops in Greiner 96-well plates which were placed in a TAP storage vault [19]. After optimisation, crystals grew in 2–7% polyethylene glycol 3350, 5 mM MgSO_4 _and 50 mM MES pH6.5. Even though the crystals were small (50 μm in the longest dimension), they were suitable for data collection.

X-ray diffraction data were collected at 100 K in-house using a Rigaku MicroMax 007 generator Cu Kα radiation source and a MAR 345 imaging plate detector (MAR Research). Crystals diffracted to a resolution limit of 3.1 Å. Images were indexed and integrated with DENZO, whilst data were merged using SCALEPACK [34]. The crystals belong to space group *P*3_1 _with unit cell lengths of *a *= *b *= 63.3, *c *= 166.4 (Å), and contained four VVTK subunits within the asymmetric unit. Detailed statistics for X-ray data collection are given in Table 1.

The VVTK crystal structure was solved by molecular replacement using MOLREP [35] with the coordinates of hTK (PDB code 1W4R) [23] as the search model. Refinement was carried out using cycles of manual rebuilding with COOT [36], followed by NCS restrained refinement using CNS [37] and REFMAC5 [38] with TLS [39]. Real space electron density averaging applied to the four molecules in the VVTK crystal asymmetric unit was carried out using the program GAP (D. I. Stuart, J. M. Grimes & J. M. Diprose, unpublished). Density for the dTTP ligand was fitted as appropriate and refined. The final refinement parameters of R_work _and R_free _were 25.7 and 28.9% respectively (Table 1). Structural superpositions were performed with SHP [40], and figures were drawn using PYMOL (DeLano, W.L. (2002) DeLano Scientific, San Carlos, CA, USA). The coordinates of VVTK together with structure factors have been deposited in the protein data bank (PDB code 2J87).

### Enzyme assays

Thymidine kinase assays were carried out in a 50 μl reaction mixture containing : 50 mM Tris_HCl pH7.6, 0.1 mg/ml BSA, 2.5 mM γ-^32 ^[P] ATP, 5 mM MgCl_2_, 5 mM dithiothreitol, and nucleoside analogs. In the assays with dThd, 2'-deoxycytidine, 2'-deoxyadenine and 2'-deoxyguanosine, 1 mM of nucleoside analog was used, in the other assays, 150 μM of nucleoside was used. The samples were incubated for 30 min at 37°C. The substrate specificity of the purified enzyme was assayed by thin layer chromatography. The autoradiography was scanned with a "Biorad Personal Molecular Image FX" and the was interpreted with "Quantity one" software also from Biorad.

## Abbreviations

TK, thymidine kinase; VVTK, vaccinia virus thymidine kinase; hTK, human type II thymidine kinase 1; UuTK, *Ureaplasma urealyticum *thymidine kinase; dThd, 2'-deoxythymidine; dTTP, thymidine 5'-triphosphate; dTD(M)P, thymidine 5'-di(mono)phosphate; (S)-MCT, (South)-methanocarbathymine; (N)-MCT, (North)-methanocarbathymine.

## Authors' contributions

KEO purified, crystallized the protein; collected and processed the diffraction data, modelled, refined and analyzed the structure. NS cloned the gene. JB carried out enzymatic studies. DKS, JB, AK coordinated the study. KEO and DKS prepared the manuscript with additional input from JB and AK. All authors read and approved the final manuscript.

## Figures and Tables

**Table 1:** Statistics for crystallographic structure determination

**Data collection details:**	
Data collection site	In house
Detector	MAR345
Wavelength (Å)	1.5418
Resolution range (Å)	30.0-3.1 (3.21-3.10)
Number of unique reflections	13636
Redundancy	3.8 (2.8)
Completeness (%)	94.8 (87.9)
Average *I*/σ(*I*)	8.5 (2.3)
Rmerge^a^	0.149 (0.494)

**Refinement statistics:**	

Resolution range (Å)	30.0-3.1 (3.18-3.1)
R-factor†(R_work_/R_free_)^b^	25.7/28.9
Number of protein atoms	5206
Average B factor (Å^2^)	42.3
Rms bond length deviation (Å)	0.012
Rms bond angle deviation (°)	1.42

Figures in brackets – outer shell data. ^a^R_merge _= Σ|I - <I>|/Σ<I>;

^b^R factor = Σ|*F*_*o *_- *F*_*c*_|/Σ*F*_*o*_.
